# ROS Modulator Molecules with Therapeutic Potential in Cancers Treatments

**DOI:** 10.3390/molecules23010084

**Published:** 2017-12-31

**Authors:** Carole Nicco, Frédéric Batteux

**Affiliations:** Department “Development, Reproduction and Cancer”, Cochin Institute, INSERM U1016, University Paris Descartes, Paris 75014, France; frederic.batteux@aphp.fr

**Keywords:** reactive oxygen species, cancer, redox status, antioxidant, SOD mimic

## Abstract

Reactive Oxygen Species (ROS) are chemically reactive chemical species containing oxygen. The redox status of a cell is function of the relative concentrations of oxidized and reduced forms of proteins, enzymes, ROS, molecules containing thiol and other factors. In the organism, the redox balance is based on the generation and elimination of ROS produced by endogenous and exogenous sources. All living organisms must maintain their redox equilibrium to survive and proliferate. Enzymatic and molecular pathways control ROS levels tightly but differentially depending on the type of cell. This review is an overview of various molecules that modulate ROS production/detoxification and have a synergistic action with the chemotherapies to kill cancer cells while preserving normal cells to avoid anticancer drugs side effects, allowing a better therapeutic index of the anticancer treatments.

## 1. Oxidative Stress

### 1.1. ROS

**Figure 1:**
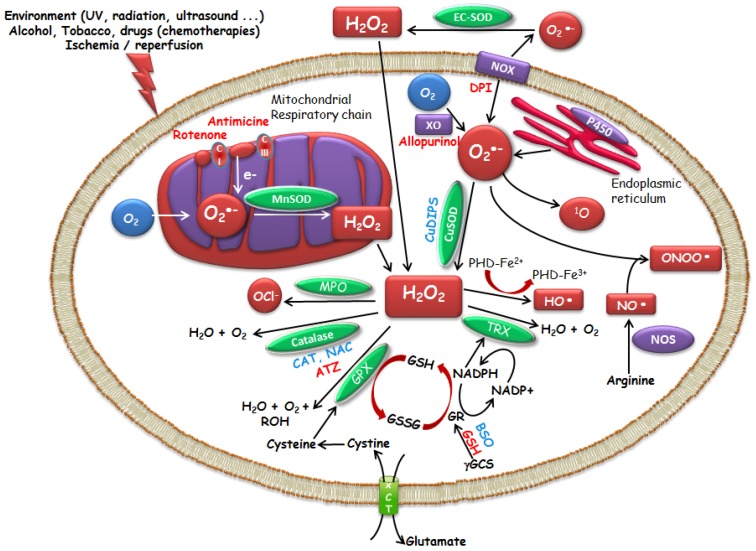
Modulators of intracellular ROS levels. Activator, **Inhibitor**. Superoxide dismutases (SOD) enzymes : Copper–Zinc-SOD (Cu–Zn-SOD, SOD1, cytoplasmic), Manganese-SOD (Mn-SOD, SOD2, mitochondrial) and Zinc-SOD (EC-SOD, SOD3, extracellular SOD). SOD mimics (Manganese (III) (4-benzoic acid) porphyrin (MnTBAP), copper (II) (3,5-diisopropylsalicylate) 2 (CuDIPS)), aminotriazol (ATZ, catalase inhibitor), buthionine sulfoximine (BSO, GSH inhibitor), catalase (CAT), reduced glutathione (GSH), glutathione disulfide (GSSG), GST (glutathione S-transferase), *N*-acetyl-l-cysteine (NAC), NADPH oxidase (NOX), NO synthase (NOS), thioredoxin (TRX), cysteine/glutamate exchanger (xCT).

Reactive Oxygen Species (ROS) are chemically reactive chemical species containing oxygen. They include free radicals like superoxide anion (O_2_•-) and the hydroxyl (•OH) radical, but also active non-radical oxygen forms, including hydrogen peroxide (H_2_O_2_) or nitroperoxide (ONOOH). As a result of normal aerobic cellular metabolism, ROS are continuously produced by eukaryotic cells. They play a key role in signaling pathways in response to intra- and extra-cellular changes. They are also permanently eliminated by several so-called anti-oxidant systems, resulting in a precise control of their intracellular concentration (Figure 1).

### 1.2. Enzymatic and Molecular Controls of ROS Levels

The redox status of a cell is function of the relative concentrations of oxidized and reduced forms of proteins, enzymes, ROS, RNS (Reactive Nitrogen Species), molecules containing thiol, and other factors. In the organism, the redox balance is based on the generation and elimination of endogenous and exogenous ROS [1]. Under physiological conditions, there is an equilibrium between intracellular ROS and endogenous antioxidants. Oxidative stress occurs when the increase in ROS breaks the redox state leading to damage to the cellular macromolecules [2,3]. There are various enzymatic systems and factors that maintain the redox status of cells. Antioxidant defense mechanisms are complex and compartmentalized allowing independent regulation of cytoplasmic, mitochondrial, and nuclear levels of ROS [4,5].

Superoxide dismutases (SOD) are intracellular enzymes, the first line of protection against free radical derivatives of oxygen. Discovered by McCord and Fridovich in 1969 [6], they catalyze the dismutation of O_2_•- in H_2_O_2_, thus preventing the coexistence of these two radical species and consequently the generation of the •OH radical by the Haber-Weiss reaction. There are three types of superoxide dismutases with different localization: Copper–Zinc-SOD (Cu–Zn-SOD, SOD1, cytoplasmic), Manganese-SOD (Mn-SOD, SOD2, mitochondrial) and Zinc-SOD (EC-SOD, SOD3, extracellular SOD) [7,8,9]. O_2_•- can be generated outside and inside the cells, in the cytoplasm and in the mitochondria. Mitochondria are the major source of O_2_•-. Superoxide is considered to be a major factor in oxidant toxicity, and mitochondrial Mn-SOD enzymes constitute an essential defense against O_2_•-. The action of SOD, however, must be coupled with that of enzymes that degrade H_2_O_2_, such as catalases or glutathione peroxidases, in order to avoid increasing concentrations of H_2_O_2_, which in the presence of iron induces the formation of •OH, one of the most toxic radicals, by the Fenton reaction. Catalase, various peroxidases, including glutathione (GSH) peroxidase and glutathione S-transferase (GST) can convert H_2_O_2_ to H_2_O.

Other enzymes perform direct detoxification of ROS. Glutathione is a tripeptide, formed by the condensation of glutamic acid, cysteine, and glycine (γ-l-Glutamyl-l-cysteinylglycine). It is represented in a simplified way by GSH (reduced form) or glutathione disulfide (GSSG) (oxidized form), the thiol function giving its main biochemical properties. GSH can remove the ROS either by a direct chemical reaction or via peroxide reduction, as the co-factor of GSH peroxidase, inducing a cycle between the reduced form and the dimerized oxidized form of glutathione [10,11]. The GSH reductase activity, which requires NADPH, allows GSH to be mainly in its reduced form in the cell. In addition, GST, a family of enzymes that covalently bind chemical reagents to GSH, contributes to the detoxification and excretion of toxic substances. The thioredoxin/thioredoxin reductase system has similar functions. Thioredoxin is a protein that acts as an antioxidant by facilitating the reduction of other proteins by formation of disulfide bridges between cysteine residues. These interactive defense mechanisms allow cells to survive in an oxidizing environment, perform the necessary biochemical processes, and use ROS/RNS as signaling molecules. Antioxidant systems, including non-enzyme low-molecular-weight antioxidants (such as, vitamins A, C, and E, polyphenols, glutathione, and coenzyme Q10) and antioxidant enzymes, fight against oxidants in cells. Countless natural products from plants [12,13], bacteria, and fungi are rich in redox active secondary metabolites and research on many of them has a long tradition and chemopreventive properties that are well established and documented [14].

The expression of antioxidant proteins is controlled by the major antioxidant response regulator Nrf2 (nuclear erythroid related factor 2), a transcription factor that regulates cellular defense responses against ROS [15]. This leucine zipper protein is widely expressed in human tissues [16,17,18] and regulates one of the most versatile adaptation mechanisms in response to cellular oxidative stress [17]. Nrf2 also plays a role in pathologic processes like cancer as it has been found to be highly constitutively expressed in some cancer cells, thereby producing a favorable environment for cancer cell survival [19,20]. Nrf2 regulates antioxidant responses by inducing the expression of genes bearing antioxidant response elements in their regulatory regions [16]. Nrf2 is ubiquitously expressed in all human organs at low constitutive levels due to tight regulation by Keap1, a substrate adaptor protein for a Cullin3-based E3 ubiquitin ligase [21,22]. Paradoxically, the transcription factor Nrf2 is known both for its role in the prevention of carcinogenesis and conversely for its involvement in the proliferation of cancer cells [15]. The overexpression of Nrf2 in cancer cells enhances drug resistance in a variety of cancers including neuroblastoma and breast, ovarian, prostate, lung, and pancreatic cancer [23,24,25].

### 1.3. Redox Equilibrum and Cellular Fate

All living organisms must maintain their redox equilibrium to survive and proliferate. Some ROS (e.g., O_2_•-, H_2_O_2_, NO) have a signaling role, therefore the balance between their generation and their detoxification by endogenous cellular defense mechanisms is essential [26,27]. Low/moderate levels of ROS are involved in normal biochemical pathways: mitogenic response, intercellular recognition, and signal transduction, immune response against infections. Physiological processes of the cell including cellular proliferation and host defense may be interrupted when the ROS exceed or antioxidants fall below the homeostatic set point. Abnormal level of ROS induces oxidative stress and damage to biological macromolecules and genotoxicity [28]. Oxidative stress is defined by excessive intracellular level of ROS [29] due to an excess of ROS production and/or a defect in antioxidant systems that can lead to cell death. The ROS excess causes potentially mutagenic DNA damage (base oxidation, strand breakage, covalent bridging) [30,31,32], lipid peroxidation [31] (which can induce apoptosis by permeabilization of mitochondrial membranes), and oxidation of proteins (e.g., activation or inhibition of protein tyrosine kinase activity with modification of intracellular signaling).

### 1.4. Redox Equilibrum and Cancer

The etiology of cancer, tumor development and its spread imply an accumulation of somatic mutations in the cells resulting in the formation of an aggregate composed of a heterogeneous cell population. In oncology, the ambivalence of ROS actions is recognized and capital. Indeed, many chemotherapies exert their cytostatic and cytotoxic effects by generating ROS. However, the production of ROS is involved in the proliferation of tumor cells either by inducing DNA lesions likely to promote the carcinogenesis process or by directly activating intracellular signals involved in the control of proliferation [7]. It is essential to understand the mechanisms underlying the ambivalence of the role of ROS in tumor cells and to use these properties for therapeutic purposes.

Numbers of detoxification mechanisms are regulated by the Nrf2 transcription factor. Under oxidative stress, ROS level is downregulated thanks to detoxification mechanisms to ensure cell survival, most of which are mediated by Nrf2. Otherwise, if the level of oxidative stress cannot be controlled, the cell is led death. In this physiological situation, Nrf2 acts as an agent to prevent ROS-induced DNA mutation, tumor transformation and carcinogenesis [33]. In a neoplastic state, Nrf2, which is normally protective, becomes deleterious. Nrf2 properties, including detoxification, are activated in cancer cells allowing their survival and growth. This can be called the “dark side of NrFf2”. Several studies conducted in humans and animals with a declared cancer report that Keap1/Nrf2 mutations or unbalanced regulation that lead to overexpression or hyperactivation of Nrf2 may participate in tumor cells proliferation. This is reported in several types of cancer like lung and pancreatic tumors [34]. The main Nrf2-dependent genes, such as HO-1 (heme oxygenase 1), NQO1 (NAD(P)H dehydrogenase quinone 1), and TrxR1 (thioredoxin reductase 1), are overexpressed and regulate cell proliferation via expression of growth factors, transmembrane receptors, protein kinases, and transcriptional regulators [35]. In addition, Nrf2 can inhibit apoptosis-regulating pathways induced by ROS in tumor cells. Indeed, there are accumulated evidence that Nrf2 activation is involved in chemoresistance of a wide number of solid cancers and leukemias. This hypothesis is supported by the observation that experimental inhibition of Nrf2 activity via in vitro RNA interference inhibits cell proliferation and increases tumor apoptosis in human pulmonary and pancreatic cancer derived lineages [23,36], while enhancing the efficacy of in vitro and in vivo chemotherapy in subcutaneous tumor models (non-small cell lung cancer or pancreatic tumor lines) [24,37]. Overexpression of Nrf2 may increase resistance to chemotherapies such as cisplatin, doxorubicin, etoposide (lung carcinoma, mammary adenocarcinoma, neuroblastoma cell lines), highlighting its likely involvement in chemoresistance phenomena [25].

Where and when the imbalance inducing oxidative stress starts is an unsolved and crucial question. But pro- and anti-oxidants can have unexpected differential effects on normal and cancer cells. Depending on their intracellular concentration and on the type and the state of cells, ROS can have either pro or anti-proliferative effects. These properties have been exploited in cancer treatment. Compounds that modulate ROS levels and kill cancer cells through the oxidative stress and, more appropriately, molecules that convert the intracellular ROS into a mix of cytotoxic chemical species that selectively kills cancer cells are already studied and are of great interest [1,38]. These compounds can act on one or few steps of the pathways we mentioned, acting directly on ROS, on the enzymes that allows ROS production or on genes that regulate those enzymes.

## 2. Therapeutic Potential of Small Molecule Catalysts with ROS Modulating Properties

### 2.1. Molecules Acting on ROS Production

In tumor cells, the basal level of ROS is higher and close to the toxic threshold. Thus, an increase in intracellular H_2_O_2_ can promote apoptosis of tumor cells and have a synergistic anti-tumor effect with that of chemotherapy.

**Figure 2:**
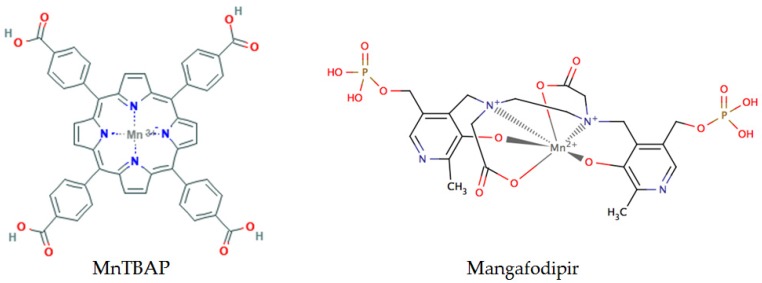
MnTBAP and mangafodipir are two Mn-SOD mimics.

SOD are ubiquitous metalloproteins that act as the first line of defense against ROS in dismutating O_2_•- to H_2_O_2_ and molecular oxygen. Currently, the main types of Mn-SOD mimics [39] include metal compounds such as Mn salen, Mn and Fe porphyrin, Mn cyclic polyamines, metal salts, metal corroles, Pt nanoparticles [40,41] and nonmetal compounds such as nitroxides, nitrones (phenyl-*tert*-butylnitrone), and pyridoxyl ethyldiamine [42]. In vitro, incubating tumor cells with anticancer drugs (taxol [43], oxaliplatin [44], or 5-FU [45]) in association with increasing concentrations of SOD mimics (MnTBAP, Figure 2, and CuDIPS) resulted in a dose-dependent increase in the cytostatic and cytotoxic effects of the chemotherapies. In vivo, murine models of subcutaneously implanted tumors have demonstrated a decrease in tumor growth under the effect of SOD mimics associated or not with chemotherapy [46,47]. SOD mimics have expanding therapeutic potential in oncology. These compounds can be used in combination with chemotherapy and radiotherapy, thus, enhancing the effectiveness of such treatments in cancer cells, while attenuating drug side-effects and toxicity issues related to radiation. In vivo models have clearly shown the beneficial effect of SOD mimics in the protection against radiation injuries [48], and improvement of the therapeutic index in anti-cancer drugs [47]. In addition, SOD mimics have shown therapeutic potential in various non tumoral situations like ischemia reperfusion injury [49], protection against chemical stress [50], septic shock [51], neuronal oxidative stress [52], diabetes [53], non-alcoholic steatosis hepatitis [54], liver transplantation [49], or inflammation [55,56]. However, the stability of the compounds, their cellular uptake, subcellular distribution, and biological transformations are factors that could override the impact and the efficacy of SOD mimics [57].

Mangafodipir (Figure 2) is a powerful SOD mimic endowed with SOD-, catalase-, and glutathione reductase–like properties. Therefore, it can target multiple steps of the ROS cascade by detoxifying O_2_•- and H_2_O_2_ and by restoring GSH stores [49,50]. This SOD mimic can protect a variety of normal cells from oxidative stress, and in particular from the oxidative stress induced by oxaliplatin, without abrogating the drug’s anticancer effect [46,47,49,50]. Tumor cells have high basal levels of O_2_•- that is further increased by oxaliplatin treatment. Co-incubation of oxaliplatin-treated tumor cells with mangafodipir transform large amount of O_2_•- into H_2_O_2_ that overwhelmed the detoxification capabilities of GSH induced by mangafodipir. By contrast, in normal cells with a low production of O_2_•- the production of H_2_O_2_ induced by oxaliplatin do not exceed the GSH concentration induced by mangafodipir. The protective effect of Mangafodipir and *N*-acetyl-l-cysteine (NAC) may derive from their ability to detoxify H_2_O_2_ via GSH activity. However, Mangafodipir unlike NAC has a synergistic effect with certain chemotherapies to diminish tumor growth because of its unique SOD mimics activity. In mice implanted with a subcutaneous tumor, the combination of paclitaxel with either mangafodipir, Cu–Zn-SOD or Mn-SOD mimics demonstrated a synergistic anti-tumor activity of these molecules. However, Cu–Zn-SOD or Mn-SOD have no protective effect on normal cells, because of their lack of GSH-reductase like activity which reduces the ability of these molecules to detoxify H_2_O_2_. Interestingly, mangafodipir prevents chemotherapy-induced leucopenia. In paclitaxel-induced leucopenia in mice, pretreatment of animal with mangafodipir not only prevents leucopenia but also the death of leucopenic animals infected with lethal inoculum of Staphylococcus Aureus [47].

Chemotherapy-induced peripheral neuropathy (CIPN) is a severe and long lasting side effect caused by diverse anticancer agents that damage sensory and/or motor nerves. CIPN occurs in 30–70% of patients treated with specific categories of anticancer agents [58]. Symptoms of CIPN include numbness, pain, burning, tingling, heat/cold hyperalgesia, and mechanical allodynia, as well as reduced motor function [59]. The chemotherapy has sometimes to be interrupted because of side effects and thus the chance of efficacy diminishes. Furthermore, when the treatment ends, CIPN can resolve in a short time period but sometimes persist as aftereffect of cancer therapy. Mangafodipir is a chelate of manganese and of the ligand fodipir, a vitamin B6 derivative. Vitamin B6 is known for its ability to maintain normal neurological functions and for its neuroprotective activity [60]. Clinical and electrophysiological tests conducted on CIPN mice models bearing tumors showed that mangafodipir can slow down the onset of locomotor and sensitivity disturbances and neuromuscular hyperexcitability in mice treated with oxaliplatin. Mangafodipir prevents oxaliplatin-induced neuropathy by scavenging O_2_•- through its SOD-like activity. In mice model, mangafodipir has both curative and preventative properties in oxaliplatin-induced neuropathy [61]. Finally, mangafodipir is endowed with numerous other effects as it abrogates ROS-mediated apoptosis/necrosis of hepatocytes in the murine model of acetaminophen-induced acute liver failure [50] and prevents lesions of ischemia-reperfusion injury of the liver [49].

In human, NACs, such as Mangafodipir, protects healthy volunteers and cancer patients’ normal leukocytes from the cytotoxicity of paclitaxel, oxaliplatin, and Fluoro-Uracil [46,47]. Moreover, mangafodipir prevents and/or relieve oxaliplatin-induced neuropathy in cancer patients [61].

**Figure 3:**
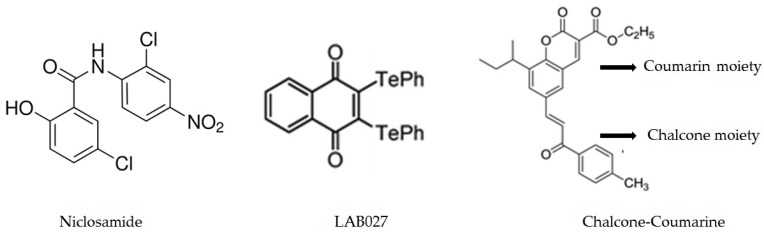
Niclosamide, organotellurides (LAB027), and chalcone-coumarine can modulate intracellular redox status.

Niclosamide (Figure 3) is an anti-helminthic with a known safety profile. It has been proven that niclosamide is an effective radiosensitizer in non-small cell lung cancer cells [62]. Niclosamide sensitize cells to H_2_O_2_, through activation of the p38 MAPK-c-Jun axis, thereby enhancing apoptosis [62]. This anti-inflammatory molecule inhibits the oxidative phosphorylating activity and has antitumor properties via the inhibition of oncogenic pathways such as STAT3, NF-κB, Wnt/β-catenin, and Notch [63]. A recent study on mice models has highlighted that niclosamide can increase the therapeutic index of oxaliplatin by both, reducing the neurodegenerative side-effects of oxaliplatin in vivo and increasing the cytotoxic effect of this chemotherapy on cancer cells in vitro and in vivo through the decrease of both H_2_O_2_ and O_2_•- productions in neurons while their levels increase in tumor cells [64].

Organotellurides (Figure 3) are well described redox-catalyst molecules with original pro-oxidative properties [65]. The ability of organotelluride catalysts to generate an intracellular oxidative burst alone and in combination with a pro-oxidative cytotoxic drug offers interesting potential and results. Selenium- and tellurium-based agents turn the oxidizing redox environment present in certain cancer cells into a lethal combination of reactive species that propel these cells over a critical redox threshold and finally kill them through apoptosis. This kind of toxicity is highly selective because normal healthy cells remain largely unaffected since their naturally low levels of oxidizing species can withstand some increase in ROS levels. Compounds that associates a quinone core along with two tellurium atoms can thus be considered as the prototype of pro-oxidative tellurium compounds [66,67]. This type of molecule can induce an overproduction of H_2_O_2_, a decrease the survival of both human and murine colon cancer cell lines in vitro. Cell death occurs by necrosis in a caspase-independent mechanism mediated by ROS and associated with mitochondrial damage and autophagy [67]. Organotellurides synergizes with oxaliplatin to kill colon cancer cell lines but not normal fibroblasts, which is a supplemental argument in favor of this kind of coupled treatment. The differential effects observed on tumor and on non-transformed cells are linked to differences in the modulation of reduced glutathione metabolism between the two types of cells. In mice grafted with tumor cells, the treatment with the organotelluride alone decreased tumor growth and synergized with oxaliplatin to further decrease tumor development. A particular organotelluride, LAB027 (Figure 3), has been described with no toxicity on leukocytes, neutrophils, and platelets, and in addition this pro-oxidant can protect against the harmful hematological effects of oxaliplatin [67]. This phenomenon is confirmed by the improved survival of E. coli-infected mice, when they are treated with LAB027, even though it is associated with chemotherapy. The effectiveness of another organotelluride in the treatment of sclerodermic mouse seems to be linked to pro-oxidative and cytotoxic effects on hyperproliferative fibroblasts [66], confirming the differential effect endowed by this type of catalyst. Nevertheless, compounds that contain quinones and tellurium are cytotoxic at low concentrations [68]. Selenium and tellurium compounds do not necessarily act via the generation of ROS, they seem to interfere with various cellular pathways, including a possible inhibition of the proteasome and DNA repair. Organic selenides are considerably more active compared to simple salts. The interference of selenium and tellurium compounds with multiple targets could provide new pathways for the development of effective antibiotic and anticancer agents which may go well beyond the traditional notion of selenium as a simple antioxidant.

Coumarins (Figure 3) a fragrant organic chemical compound belonging to the benzo-alpha-pyrones family, an important class of compounds that present diverse pharmacological properties, such as anti-HIV [69], antibacterial [70], and antioxidant [71] has been extensively investigated for their cytotoxic properties against cancer cell lines [72]. Some promising coumarin–chalcone hybrids (Figure 3) have also been tested in vitro and in vivo in a murine model of colon cancer previously described and presented interesting anticancer parameters [72,73].

**Figure 4:**
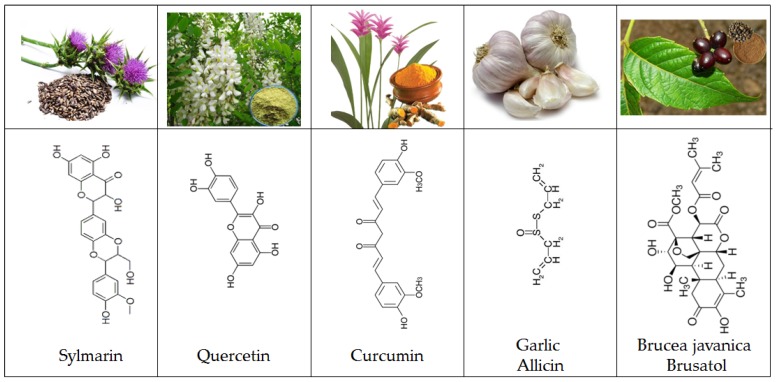
Sylmarin, quercetin, curcumin, allicin and brusatol are natural products that can modulate intracellular redox status.

Among the most famous dietary antioxidants such as berries and spices is the flavone family. Recently silymarin (Figure 4), a plant flavonoid, showed a strong cell cycle arrest and interaction with some cell cycle regulator-cyclins such as cell division cycle (Cdc25) phosphates A, B, and C [74]. These key actors in eukaryotic cell cycle control are overexpressed in various primary tumor cells [74]. Quercetin (Figure 4) is one of the most active flavonoids and many medicinal plants own their effectiveness to their high concentration in this molecule. Various pharmacological activities have been attributed to this phytochemical including antioxidant, anti-inflammatory, anti-microbial, and anti-allergic properties as well as chemopreventive, anti-genotoxic, and anti-tumor activities [75,76,77]. Quercetin can suppress the initiation, growth, and dissemination of induced tumors in animal models [75]. It can inhibit cell growth and induce apoptosis, necrosis [78], and autophagy in cancer cells [75,78]. Among flavonoids, quercetin is known as a free radical-scavenging antioxidant [79]. It has been shown to protect gastric epithelial cells against oxidative damage due to its antioxidant activity as ROS scavenger and metal chelator [79,80]. In addition to its antioxidant activity, quercetin exerted an anti-proliferative effect in gastric cancer cell line pro-apoptotic, mainly through induction of apoptosis [81].

Curcumin (Figure 4) is the principal curcuminoid of turmeric (Curcuma longa), a member of the ginger family. This polyphenol exhibits anti-angiogenesis, anti-proliferation, anti-invasion, anti-metastasis, and apoptosis [82]. Curcumin induced ROS production results in autophagic activation and concomitant cell death in HCT116 human colon cancer cell [83]. Curcumin acts as a growth inhibitor for H. pylori and is remarkably nontoxic and is well tolerated by humans at doses up to 12 g/day [84]. It has a poor bioavailability and many efforts have been performed to increase its bioavailability [85]. Curiously, despite this apparently poor bioavailability, its effects are not limited to the gastrointestinal tract, but occur in many organs, including the brain [86].

Garlic (*Allium sativum* L.) has been used as a spice and medicinal plant since ancient times. Garlic produces the thiol-reactive defense substance, allicin (Figure 4). This reactive sulfur species has oxidizing properties [87], and is able to oxidize thiols in cells, e.g., glutathione and cysteine residues in proteins. A more oxidized glutathione pool leads to a higher cellular redox potential. This organosulfur induces apoptosis by enhancing the level of mitochondrial cytochrome c and Bax release. Allicin exhibits numerous biological potentials such as anti-oxidant (81), anti-microbial, and anti-carcinogenic activities [88,89]. This compound is able to inhibit the proliferation and survival of numerous tumors [90,91] including colon, lung, cervix, breast, and gastric cancer. It has been shown that Allicin can reduce cell viability and cell proliferation in a concentration dependent manner, through glutathione oxidation [92]. Different cell lines (human lung epithelium carcinoma (A549), mouse fibroblast (3T3), human umbilical vein endothelial cell (HUVEC), human colon carcinoma (HT29), and human breast cancer (MCF7) cell lines) differed in sensitivity to allicin in regard to viability, cell proliferation and glutathione oxidation. The 3T3 and MCF-7 cells showed a higher proportion of apoptosis compared to the other cell types. Allicin like the two previously described compounds has low bioavailability that requires in high doses and limits their application [93].

### 2.2. Compounds Acting on Nrf2 (Brusatol, DMF)

Compounds that can modulate the Nrf2 pathway and enhance the efficacy of chemotherapy are of prime interest. 

**Figure 5:**
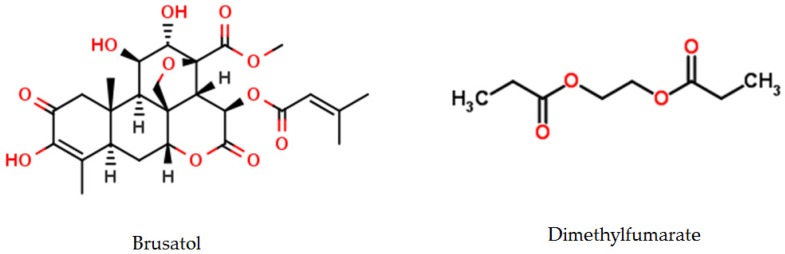
Brusatol and Dimethylfumarate are two Nrf2 modulators.

Brusatol (Figure 4 and Figure 5), known as Ya-Dan-Zi in Chinese, is the dried ripe fruit of Brucea javanica. This molecule is traditionally used for the treatment of dysenteric disorders, malaria and tumors, and it is known as a rich source of quassinoids [94,95]. The anti-inflammatory [96,97], pro-apoptotic [98], anti-metastatic [99] properties of brusatol, and its ability to reverse antineoplastic drug resistance [100,101] and radiation resistance [102], indicate the potential of brusatol to be used in the treatment of inflammatory and neoplastic diseases. In vitro and in animals models, it has been shown that Brusatol enhances chemotherapy efficacy in lung and endometrial cancers by specifically inhibiting the Nrf2 pathway [100,103]. Recent experimental data have shown that brusatol inhibits cellular growth and induces apoptosis in pancreatic cancer cell lines [104,105]. In pancreatic cancer, brusatol inhibits tumor growth by modulating oxidative stress and inducing apoptosis [106,107]. A recent study provided justifications for conducting clinical trials in the future to evaluate the safety and efficacy of this natural product in patients with pancreatic cancer. This study is consistent with previous reports on the efficacy of brusatol in various cancers [97,99,101,102,103], some of which included PDAC cell lines [104,105].

In human, brusatol combined with chemotherapy improved quality of life in a cohort of non-small cells lung cancer patients without any increase in toxicity [108], but further investigations need to be done.

Dimethylfumarate (DMF, Figure 5) is a derivative of fumaric acid used in order to activate the Nrf2 pathway. This molecule induces a covalent modification of the thiols in some cysteine residues of KEAP1 resulting in conformational changes of KEAP1, ultimately inducing a disturbance of the KEAP1-Nrf2 interaction. Thus, DMF allows a stabilization of Nrf2 that migrates into the nucleus where it activates its target genes to exert its antioxidant effects. DMF is also emerging as a key component of the transduction machinery to maintain proteostasis [109]. Several studies have shown cytoprotective and antioxidant effects of DMF in non-cancer models (56–59), which appeared related to the induction of the Nrf2 pathway (55–58). This molecule could limit to an acceptable level the accumulation of ROS produced in excess by the mitochondrial respiratory chain of hypermetabolic and proliferative cancer cells. Nevertheless, overexpression of the NRF2 protein has also recently emerged as a potential mechanism of resistance to platinum and other cytotoxic drugs [110,111,112]. DMF has a dose-dependent effect in cancer cells. It is cytoprotective at lower concentrations by inducing NRF2 but at higher concentrations it inhibits the NRF2 stabilizer DJ-1, which in turn inhibits NRF2 activation, induces ROS, and subsequently promotes cancer cell death. These findings implicate the effect of DJ-1 on NRF2 in cancer development and identify DMF as not only an activator, but also an inhibitor of both NRF2 and DJ-1, which may be useful in exploiting the therapeutic potential of these endogenous antioxidants. The antitumoral effects of DMF has been reported in various mouse models at a dose well tolerated by humans and applicable to clinical practice [113]. These observations could be related to DMF tissue accumulation, which is associated with longer treatment or alternatively, to a lesser DMF toxic effect on non-cancer cells compared with the malignant ones [114]. This differential effect of DMF may be related to the lower level of oxidative stress in normal cells that render them more permissive to NRF2 depletion. In addition, previous report showed that activation of the NRF2 antioxidant response pathway is independent of DJ-1 in primary neural cells and tissues, suggesting that the protective role of DJ-1 may be less important for NRF2 function in non-cancer cells [115]. In a syngenic tumor mouse model daily administration of DMF alone or associated with paclitaxel can attenuate the protein levels of both NRF2 and its stabilizer DJ-1 [114]. The significant increase of advanced oxidized protein products in the sera of DMF-treated mice indicates that DMF anti-tumoral effect can be linked to systemic oxidative stress [114]. The cytotoxic effect of DMF against several tumor cell lines has been demonstrated in vitro [116,117,118,119] and the induction of oxidative stress as well [120,121].

## 3. Conclusions

Reactive oxygen species are natural byproducts of the normal cellular metabolism. They are implicated in various signaling pathways in response to intra- and extra-cellular changes and are also permanently eliminated by several so-called anti-oxidant systems, resulting in a precise control of their intracellular concentration. Increased levels of ROS either because of their overproduction or lack of detoxification can lead to cell death through alteration of DNA, proteins, or lipids. This cytotoxic property of ROS has been exploited for years to kill cancer cells and numerous anti-cancer chemotherapies exert their therapeutic properties at least in part through overproduction of ROS. More recently, the possibility to modulate ROS production/detoxification through the use of small molecules with redox modulating properties has emerged as synergized with the chemotherapies to kill cancer cells while preserving normal cells to avoid anticancer drugs side effects. Understanding more clearly the mechanism of ROS metabolism in normal and in various type of cancer cells along with the discovery of additional natural or chemical molecules with redox properties is a real challenge for the future of anticancer treatment. These small molecules can be considered as prospective drugs to overcome classical resistance in cancer cells while preserving normal cells.

## Abbreviations

3T3	mouse fibroblast
5-FU	5-Fluoro-Uracile
A549	human lung epithelium carcinoma
ATZ	aminotriazol
BSO	buthionine sulfoximine
CAT	catalase
CuDIPS	copper (II) (3,5-diisopropylsalicylate) 2
Cu-Zn-SOD	Copper-Zinc-SOD
EC-SOD	Zinc-SOD, Zn-SOD
GSH	reduced glutathione
GSSG	glutathione disulfide, oxidized GSH
GST	glutathione S-transferase
H_2_O_2_	hydrogen peroxide
HO-1	heme oxygenase 1
HT29	human colon carcinoma
HUVEC	human umbilical vein endothelial cell
Keap1	Kelch-like ECH-associated protein
MCF7	human breast cancer
Mn-SOD	Manganese-SOD
MnTBAP	Manganese (III) tetrakis (4-benzoic acid) porphyrin
NAC	*N*-acetyl-l-cysteine
NOX	NADPH oxidase
NQO1	NAD(P)H dehydrogenase quinone 1
Nrf2	nuclear erythroid related factor 2
O_2_•-	superoxide anion
•OH	hydroxyl radical
ONOOH	nitroperoxide, peroxynitrous acid
ONOO-	peroxynitrite
NOS	NO synthase
RNS	Reactive Nitrogen Species
ROS	Reactive Oxygen Species
TRX	thioredoxin
TrxR1	thioredoxin reductase 1
Xct	cysteine/glutamate exchanger
